# Space Flight Effects on Antioxidant Molecules in Dry Tardigrades: The TARDIKISS Experiment

**DOI:** 10.1155/2015/167642

**Published:** 2015-01-13

**Authors:** Angela Maria Rizzo, Tiziana Altiero, Paola Antonia Corsetto, Gigliola Montorfano, Roberto Guidetti, Lorena Rebecchi

**Affiliations:** ^1^Department of Pharmacological and Biomolecular Sciences, Università degli Studi di Milano, Via D. Trentacoste 2, 20134 Milano, Italy; ^2^Department of Education and Human Sciences, University of Modena and Reggio Emilia, Via A. Allegri 9, 42121 Reggio Emilia, Italy; ^3^Department of Life Sciences, University of Modena and Reggio Emilia, Via G. Campi 213/D, 41125 Modena, Italy

## Abstract

The TARDIKISS (Tardigrades in Space) experiment was part of the Biokon in Space (BIOKIS) payload, a set of multidisciplinary experiments performed during the DAMA (Dark Matter) mission organized by Italian Space Agency and Italian Air Force in 2011. This mission supported the execution of experiments in short duration (16 days) taking the advantage of the microgravity environment on board of the Space Shuttle Endeavour (its last mission STS-134) docked to the International Space Station. TARDIKISS was composed of three sample sets: one flight sample and two ground control samples. These samples provided the biological material used to test as space stressors, including microgravity, affected animal survivability, life cycle, DNA integrity, and pathways of molecules working as antioxidants. In this paper we compared the molecular pathways of some antioxidant molecules, thiobarbituric acid reactive substances, and fatty acid composition between flight and control samples in two tardigrade species, namely, *Paramacrobiotus richtersi* and *Ramazzottius oberhaeuseri*. In both species, the activities of ROS scavenging enzymes, the total content of glutathione, and the fatty acids composition between flight and control samples showed few significant differences. TARDIKISS experiment, together with a previous space experiment (TARSE), further confirms that both desiccated and hydrated tardigrades represent useful animal tool for space research.

## 1. Introduction

As the interest in space exploration grows, it becomes of great importance to predict and know the response of uni- and multicellular organisms to unfavourable space conditions, including microgravity. This allows us to elaborate the opportune countermeasures to avoid the risks imposed by space environmental stressors. To date many studies for understanding physiological, biochemical, and molecular mechanisms against space stressors are performed on unicellular organisms or cultivated cells of multicellular organisms [1]. Although the experiments on cell cultures are useful, it is equally clear that cell cultures represent only the first level of life organization and they cannot be compared to the response of an entire multicellular living organism. The use of animals in space research allows us to conduct experiments with organisms characterized by a high level of hierarchical biological complexity and physiological processes, comparable to those of humans [2].

Even though animals could be useful models in space research, their use is often limited by the fact that many of them need specific rearing bioreactors of large volume [1, 3]. Tardigrades, or water bears, are little known and neglected animals that allow overcoming this problem. Their use in space research is supported by several reasons: (i) they are miniaturized animals (from 200 to 1000 *μ*m in length) that can be kept and reared in small facilities/bioreactors; (ii) while having tissues and organs, they are simpler than several other animals, having a limited cell number (about 1000); (iii) they can be easily reared under lab conditions; (iv) many of them are parthenogenetic, often apomictic, so clonal lineages can be obtained [1, 2]. Although all tardigrades are aquatic animals, they thrive in terrestrial habitats subjected to periodic desiccation thanks to their ability to enter a highly stable state of suspended metabolic activity called anhydrobiosis [4]. Entering in this physiological state, tardigrades lose up to 97% of their body water and shrivel into a desiccated structure about one-third of its original size. When rehydrated, tardigrades can return to their active metabolic state in a few minutes to a few hours [4, 5]. Desiccated tardigrades can persist in anhydrobiosis for several years, and a remarkable resilience to physical and chemical extremes has been documented [4–6]. By possessing the abilities to withstand complete desiccation, severe cold, microgravity, vacuum, and high levels of ionizing and UV radiations, anhydrobiotic tardigrades fulfill the most important criteria for tolerating exposure to natural space conditions including open space [2].

Tardigrades have already been exposed to space stressors on Low Earth Orbit during the FOTON-M3 mission in 2007 with different projects (TARDIS [7]; TARSE [1, 8]; RoTaRad [9]). With the TARSE (Tardigrade Resistance to Space Effect) project, we analyzed the responses of both desiccated and hydrated physiological state of the tardigrade* Paramacrobiotus richtersi* to spaceflight conditions within the spacecraft [1, 8]. Microgravity and radiation had no effect on animal survival and life history traits even though a higher number of laid eggs, a shorter egg development time, and a higher number of flight-born juveniles were recorded with respect to tardigrades reared on Earth [1, 10]. In addition, spaceflight induced in active tardigrades an increase of glutathione content, an increase of glutathione peroxidase activity, and a decrease of catalase, superoxide dismutase, and glutathione reductase activities [1]. Lastly, no change in thiobarbituric acid reactive substances was detected. On the basis of these results, we developed the new project TARDIKISS (Tardigrades in Space), with the aim to deepen the study of survivorship, life history traits, and regulation of antioxidant defences on alive desiccated tardigrades under space stressors including microgravity exposure. The flight tardigrades of the project TARDIKISS have had a very high survival (more than 91%) and females laid eggs which were able to hatch producing normal newborns able to reproduce in adulthood [11]. In this paper we compared the molecular pathways of molecules with antioxidant activity, thiobarbituric acid reactive substances, and fatty acid composition between flight tardigrades and ground control ones with the final aim to provide news about the biochemical mechanisms underlying resistance to space stress conditions.

## 2. Material and Methods

### 2.1. TARDIKISS Project

The TARDIKISS project was part of the BIOKIS (Biokon in Space) payload: a set of multidisciplinary experiments in the field of biology and dosimetry performed in microgravity condition during the DAMA (Dark Matter) mission organized by Italian Space Agency (ASI) and Italian Air Force in 2011. This mission supported the execution of experiments in short duration (16 days) taking the advantage of the microgravity environment on board of the last mission (STS-134) of Space Shuttle Endeavour docked to the International Space Station (ISS) [11].

TARDIKISS was composed of three sample sets: one flight sample (F) and two ground control samples. The former control (temperature control, TC) was a postflight control in which samples were exposed to the temperature profile experienced by tardigrades the days immediately before, during, and just after the flight mission; the latter (laboratory control, LC) was maintained in Modena laboratory for the duration of the flight at constant temperature. These samples provided the biological material used to test as space stressors, including microgravity, affected animal survivability, life cycle, DNA integrity, and changes of the pathways of molecules working as antioxidants.

**Figure 1:**
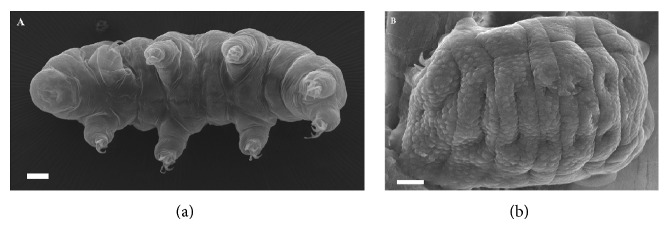
Micrographs by scanning electron microscopy of the tardigrade* Ramazzottius oberhaeuseri* showing its two physiological states. (a) Hydrated and metabolically active specimen. (b) Desiccated and metabolically inactive specimen. Bars: a = 10 *μ*m; b = 5 *μ*m.

Two anhydrobiotic eutardigrade species were considered, namely,* Paramacrobiotus richtersi* (Murray, 1911) (Macrobiotidae) and* Ramazzottius oberhaeuseri* (Doyère, 1840) (Ramazzottiidae).* Paramacrobiotus richtersi* is the model species already used in the FOTON mission [1].* P. richtersi* was extracted from a hazel leaf litter (sample code C3499); it is carnivorous, white in colour, and the population here considered is bisexual and amphimictic.* R. oberhaeuseri* (Figure 1) was extracted from the lichen* Xanthoria parietina* (L.) Th. Fr. (1860) (sample code C3282); it is herbivorous, brown/red in colour, and the population considered in this study is unisexual and parthenogenetic. To extract tardigrades from their substrates, leaf litter and lichen were sprinkled with tap water and after 15 min submerged in water for 15 min at room temperature. Later, each substrate was sieved (mesh size of sieves: 250 *μ*m and 37 *μ*m) under running water; then animals were picked up from the sieved sediments with a glass pipette under a stereomicroscope.

For both tardigrade species, animals in desiccated (anhydrobiotic) physiological state were used. To obtain desiccated specimens, tardigrades were dehydrated in lab under controlled air relative humidity (RH) and temperature. After extraction from their substrates, tardigrades were kept in water for 24 h at 15°C without any food source. Then, they were forced into anhydrobiosis by placing groups of animals on a square (1 cm^2^) blotting paper with natural mineral water (30 *μ*L). The paper with tardigrades was initially exposed to 80% RH and 18°C for 4 h, then to 50% RH at 18°C for 4 h in a climatic chamber, and finally to 0–3% RH at room temperature for 12 h [1].

Papers with desiccated tardigrades were stored in twelve small plastic Petri dishes (1.8 cm × 1.0 cm) enveloped with parafilm and integrated within the Biokon facility (Kayser Italia), where a radiation dosimeter for neutrons and* i*-button data logger recorded temperature were also present [11]. During the entire flight mission the temperature profile was relatively constant ranging from 21°C to 25°C [11], while the dose equivalent rates due to space radiation exposure were 320 *μ*Sv (measured by TLD 100 and TLD 700) and 360 *μ*Sv (measured by TLD 600) [11].

### 2.2. Biochemical Assays

Biochemical assays were performed on desiccated tardigrades comparing F samples with TC samples.

The activities of the enzymes superoxide dismutase (SOD total activity), catalase (CAT), glutathione peroxidase (GPx), and glutathione reductase (GR) were evaluated. The total glutathione (GSH) content, thiobarbituric acid reactive substances (TBARS), and fatty acid composition were also determined as previously described [12].

Substrates and reagents for enzyme determinations were NAD(P)H, DTNB, GSH, GSSG, glutathione reductase, and tert-butyl hydroperoxide; all of them were purchased from Sigma-Aldrich (St. Louis, Missouri, USA). For each sample set and each species, 6 or 8 (with the exception of SOD) replicates each made up by 10* in toto* tardigrades were homogenized in water, on ice, with potter using 3 cycles of 30 sec each. The homogenate was assayed for protein content (according to [13]) and used for enzyme determination. For each enzyme, homogenates were analyzed in duplicate.

Briefly, the activity of the enzyme superoxide dismutase was assayed using the method based on NAD(P)H oxidation inhibition (according to [14]); the inhibition of NADPH oxidation by superoxide, which was chemically generated, was measured at 340 nm for 20 min, in the presence of tissue extracts. The incubation mixture included 213 *μ*L of TDB (triethanolamine/diethanolamine 100 mM, pH 7.4), 10 *μ*L of NADPH 7.5 *μ*M, 7 *μ*L of EDTA-MnCl_2_ (100 mM–50 mM), and 20 *μ*L of sample or blank. One unit of SOD activity was defined as the amount of enzyme required to inhibit the rate of NADPH oxidation by 50%.

To evaluate the activity of catalase, samples were assayed by measuring the consumption of H_2_O_2_ (according to [15]). Consumption of hydrogen peroxide by the tissue extracts was determined at 240 nm for 1 min at 30°C. The incubation mixture included 10 *μ*L of H_2_O_2_ 200 mM, 20 *μ*L of homogenate, and 170 *μ*L of Na-phosphate buffer (50 mM, pH 7.0). One unit of CAT activity was defined as the amount of enzyme required to catalyze the decomposition of 1 mmol of H_2_O_2_ min^−1^.

The activity of the glutathione reductase was assayed following the oxidation of NADPH (according to [16]). Briefly, GSSG reduction and NADPH consumption were followed at 340 nm. The incubation mixture included 5 *μ*L of GSSG 125 mM, 3 *μ*L of NADPH 11 mM, animal homogenate from 20 to 50 *μ*L, and K-phosphate buffer (100 mM, pH 7.0) to reach a final volume of 0.25 mL. One unit of GR activity was defined as the amount of enzyme required to catalyze the oxidation of 1 *μ*mol NADPH min^−1^.

To evaluate the activity of selenium-dependent glutathione peroxidase, the enzyme activity was assayed (according to [17]) following the decrease in the absorbance at 340 nm for 3 min, which corresponds to the rate of GSH oxidation to GSSG in the presence of NADPH and glutathione reductase. The incubation mixture included 5 *μ*L of GSH 100 mM, 3 *μ*L of NADPH 22 mM, GR 1 unit, 5 *μ*L of tert-butyl hydroperoxide 20 mM, from 20 to 50 *μ*L of animal homogenate, and EDTA-K phosphate buffer (3 mM–100 mM pH 7.0) to reach a final volume of 0.25 mL. One unit of GPx activity was defined as the amount of enzyme required to catalyze the oxidation of 1 *μ*mol of NADPH min^−1^.

To measure the total glutathione, tardigrades were homogenized on ice in 5% metaphosphoric acid; the homogenate was centrifuged at 5000 ×g for 10 min at 4°C, and the supernatant was assayed (according to [18]) with some slight modifications. Briefly, the sulfhydryl group of GSH, also generated from GSSG by adding GR, reacts with DTNB (5,5′-dithiobis-2-nitrobenzoic acid) and produces a yellow-coloured 5-thio-2-nitrobenzoic acid (TNB). The rate of TNB production is directly proportional to this reaction, which in turn is directly proportional to the concentration of GSH in the sample. The measurement of the absorbance of TNB at 412 nm provides an accurate estimation of the GSH level present in the sample.

To evaluate the thiobarbituric acid reactive substances (TBARS), tardigrade samples, standards (from 2.5 to 100 pmol TEP, 1,1-3,3 tetraethoxypropane), and blanks were assayed (according to [19]), both before and after induction of lipid peroxidation by FeSO_4_ and ascorbic acid. TBARS were determined using a fluorescence spectrophotometer (Carly Eclipse, Varian, CA USA) at an excitation wavelength of 517 nm and an emission wavelength of 550 nm. For each sample set (F and TC) and species (*R. oberhaeuseri* and* P. richtersi*) 2 or 4 replicates were analyzed.

To evaluate the fatty acid composition, lipids were extracted from groups of 10 desiccated tardigrades with chloroform/methanol (according to [20]). The total extract was used for derivatization with sodium methoxide in methanol 3.33% w/v to obtain the fatty acid methylesters (FAME). FAME were injected into a gas chromatograph (Agilent Technologies 6850 Series II) equipped with a flame ionization detector (FID) under the following experimental conditions: capillary column: AT Silar length 30 m, film thickness 0.25 *μ*m: gas carrier: helium: temperatures: injector 250°C, detector 275°C, oven 50°C for 2 min, and rate of 10°C min^−1^ until 200°C for 20 min. A standard mixture containing methyl ester fatty acids was injected for calibration. For each sample set and species 2 or 4 replicates were analyzed.

### 2.3. Statistical Analysis

Data were analyzed with Mann-Whitney test and expressed as mean ± SD using the programme SPSS.

## 3. Results

**Figure 2:**
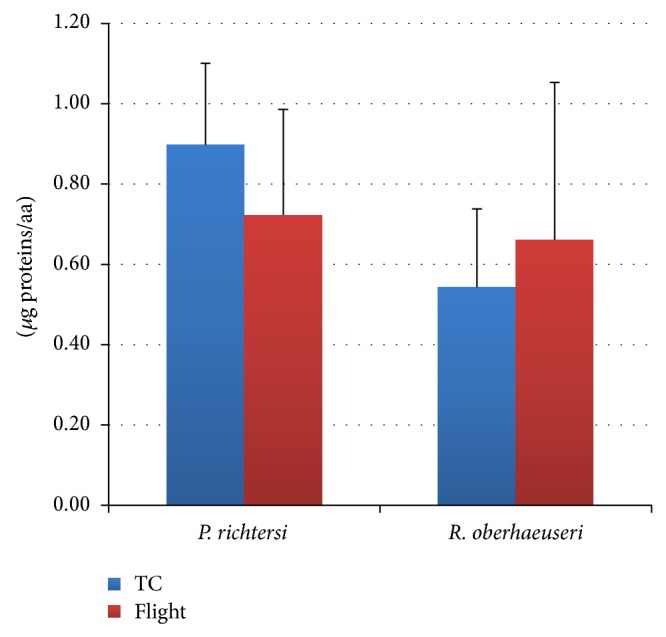
Total protein content in flight and ground temperature control (TC) samples in the tardigrades* Paramacrobiotus richtersi* and* Ramazzottius oberhaeuseri*. The bars show the mean with SD.

The results of the enzyme activities in the tardigrades* Paramacrobiotus richtersi* and* Ramazzottius oberhaeuseri* are always indicated in relation to *μ*g of proteins. It is worth noting that* R. oberhaeuseri* contains a lower amount of proteins compared to* P. richtersi* (Figure 2).

**Figure 3:**
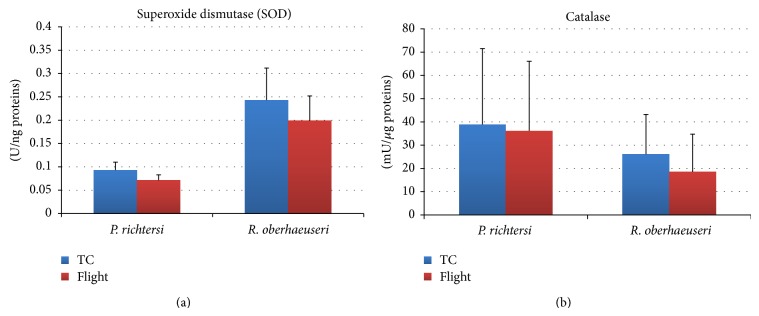
Superoxide dismutase (a) and catalase (b) activities in flight and ground temperature control (TC) samples in the tardigrades* Paramacrobiotus richtersi* and* Ramazzottius oberhaeuseri*. The bars show the mean with SD.

**Figure 4:**
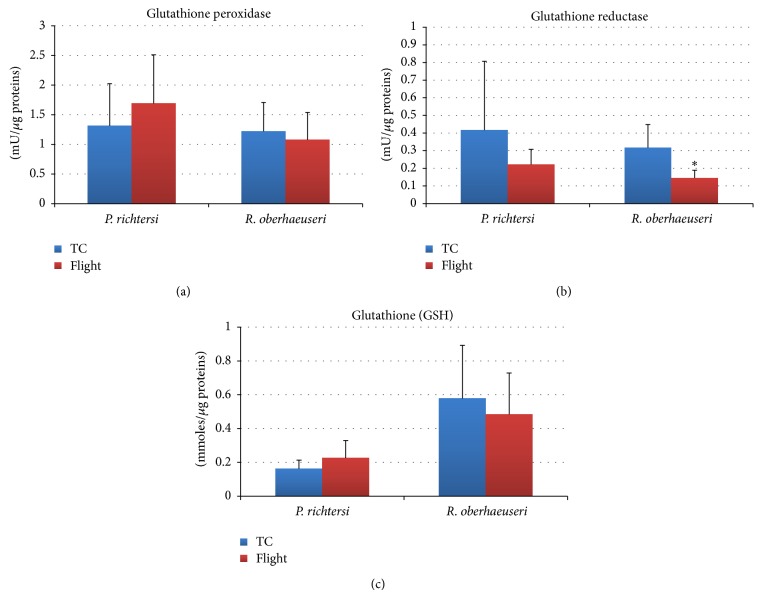
Glutathione peroxidase (a), glutathione reductase (b), and total glutathione content (c) in flight and ground temperature control (TC) samples in the tardigrades* Paramacrobiotus richtersi* and* Ramazzottius oberhaeuseri*. The bars show the mean with SD; ^*^ *P* < 0.05.

In both species, the comparative analysis of the enzyme activities and other antioxidant molecules between flight (F) and temperature control samples (TC) showed few significant differences (Figures 3 and 4). In particular, a significant decrease (*P* < 0.05) of the glutathione reductase activity was detected in* R. oberhaeuseri* F samples with respect to TC samples (Figure 4(b)). Although not statistically supported, in this species a tendency to decrease catalase, superoxide dismutase, and glutathione peroxidase activity and in glutathione content was detected. In* P. richtersi*, a tendency to decrease catalase, superoxide dismutase, and glutathione reductase activities and to increase the glutathione peroxidase activity was detected. Noteworthy, differences were recorded in the activities of ROS scavenging enzymes between the two species.

The total percentage fatty acid composition of F and TC samples is reported in Table 1. In* R. oberhaeuseri* a significant decrease (*P* < 0.05) was recorded for the fatty acid C22-6 n-3 and polyunsaturated fatty acids (PUFA) in the F samples with respect to the TC samples. Moreover,* R. oberhaeuseri* has significantly lower amount of C22-6 n-3 compared to* P. richtersi*. The amount of thiobarbituric acid reactive substances (TBARS) in tardigrades, both before and after induction of peroxidation* in vitro,* is also reported in Table 1. No differences were detected between F and TC samples in both species for basal levels and after induction of peroxidation.

## 4. Discussion

Exposure to space stress conditions induces oxidative stress. Oxidative stress resulting from an imbalance between the excessive production of reactive oxygen species (ROS) and limited action of antioxidant defences is implicated in the development of many important human pathologies including atherosclerosis, hypertension, inflammation, cancer, Parkinson, and Alzheimer diseases [21]. Oxidative stress may be highly destructive also in anhydrobiotic organisms even if the lower cellular water content decreases the production of ROS [21, 22]. Under normal conditions, antioxidant systems minimize the adverse effects caused by ROS, but desiccation stress could cause the loss or reduction of these defence control mechanisms since the metabolic activity is absent or reduced [21–24].

The ability of some animals, tardigrades among them, to survive extreme desiccation involves a complex array of factors working at structural, physiological, and molecular level. From a molecular/biochemical point of view, anhydrobiotic organisms synthesize molecules working as bioprotectants during entering, permanence, and leaving in a desiccated state [25]. For example, trehalose and sucrose stabilise the biological membrane avoiding protein unfolding and membrane disturbances; late embryogenesis abundant proteins and heat shock proteins work as chaperone systems repairing or eliminating damaged molecules, while antioxidant molecules counteract the negative effects of oxidative stress [25].

Since it is known that both hydrated and desiccated tardigrades have a good natural capability to overcome oxidative stress [26], they have been used in TARDIKISS experiments to evaluate the role of antioxidant defence in overcoming oxidative stress induced by exposure to space stress conditions such as ionizing and UV radiations.

The first space experiment (TARSE) conducted with hydrated starved specimens of the tardigrade* P. richtersi* demonstrated that some of the enzymes involved in antioxidant defences were significantly influenced by the flight stresses [1]. In particular, there was a significant decrease in catalase and SOD activities, the more active enzymes in* P. richtersi*. In addition, the glutathione system, the less active system in not stressed specimens of this species [26], was significantly induced during space flight [1]. These results could be related to the stresses experienced by the hydrated and metabolically active animals (microgravity, starvation, and radiations) during the flight. On the contrary the analysis of antioxidant defences in desiccated tardigrades of the TARDIKISS experiment showed fewer differences related to space flight even if the tendency was similar to that recorded in hydrated metabolically active animals of the TARSE experiment. A similar trend between TARSE and TARDIKISS experiments was also detected in regard to tardigrade survival since flight animals did not show significant differences in survival from temperature laboratory control ones [1, 11]. Only in* R. oberhaeuseri* (TARDIKISS experiment) a significant decrease in survival rate was recorded between F and TC samples, the species in which a significant decrease of the C22:6 n-3 fatty acid and of glutathione reductase activity and, even though not significant, of the activity of the other ROS scavenging enzymes were detected.

In conclusion, TARDIKISS experiment, together with previous space experiments using tardigrades [1, 7–9], further confirms that both desiccated and hydrated physiological states of tardigrades represent useful animal tool for space research. To further develop the space research using tardigrades, the setup of experiments with the possibility to change the exposition condition of metabolically hydrated animals, as well as the possibility to expose desiccated tardigrades to open space, is necessary. Experiments under true space condition provide a realistic evaluation of the mechanisms that could allow multicellular organisms, including tardigrades, to survive the combined and synergic effects of space stressors. Nevertheless, experiments on ground using simulators of microgravity, radiation, temperature, and other space stresses are an essential part of space research complementing experiments under true space conditions. The comparisons of two different sets of data (ground and space data) will allow better understanding of the physiological and molecular pathways of living organisms under space environment.

## Figures and Tables

**Table 1:** Percentage of fatty acid composition in the tardigrades *Paramacrobiotus richtersi* and *Ramazzottius oberhaeuseri. *

Fatty acid	*Paramacrobiotus richtersi*		*Ramazzottius oberhaeuseri*	
	TC	F	TC	F
C16:0	28.86 (1.56)	29.41 (3.53)	29.65 (1.84)	32.64 (1.15)
C16:1	8.44 (1.98)	8.91 (0.79)	6.56 (1.68)	9.77 (0.23)
C18:0	14.53 (2.68)	17.86 (4.87)	16.22 (1.76)	18.56 (4.55)
C18:1	19.85 (3.43)	17.13 (4.55)	21.45 (1.71)	20.04 (6.21)
C18:2 n-6	9.75 (3.24)	13.18 (2.99)	12.11 (6.59)	12.97 (1.69)
C18:3 n-3	2.61 (2.05)	2.25 (1.69)	4.03 (3.48)	1.50 (0.30)
C20:3 n-6	1.11 (0.89)	1.03 (0.67)	0.22 (0.15)	0.24 (0.32)
C20:4 n-6	9.78 (7.56)	5.05 (5.20)	5.87 (1.74)	2.91 (2.02)
C20:5 n-3	1.17 (0.46)	1.99 (0.78)	1.30 (1.55)	0.57 (0.77)
C22:5 n-3	0.51 (0.51)	0.14 (0.19)	0.14 (0.23)	0.23 (0.33)
C22:6 n-3	4.00 (1.27)	3.03 (0.36)	2.45 (0.36)	0.56 (0.60)^*^
PUFA	28.92 (7.49)	26.68 (4.45)	26.12 (3.25)	18.99 (0.73)^*^
TBARS basal (pmoles/*µ*g proteins)	2.81 (1.04)	2.51 (0.55)	2.77 (0.56)	2.60 (1.08)
TBARS induced (pmoles/*µ*g proteins)	26.06 (3.65)	28.25 (1.27)	43.65 (1.61)	32.91 (2.58)

TC = ground temperature control samples; F = flight samples; PUFA = polyunsaturated fatty acids; TBARS = thiobarbituric reactive substances; ^*^ *P* < 0.05; in brackets SD.
